# CU06-1004 inhibits the progression of chronic colitis and colitis-associated colorectal cancer by suppressing inflammation

**DOI:** 10.3389/fphar.2025.1684870

**Published:** 2025-10-22

**Authors:** Dongyeop Kim, Yeomyeong Kim, Haiying Zhang, Ye-Seul Kim, Minyoung Noh, Cho-Rong Bae, Young-Guen Kwon, Sang-Jun Ha

**Affiliations:** ^1^ Department of Biochemistry, College of Life Science and Biotechnology, Yonsei University, Seoul, Republic of Korea; ^2^ Department of Bio Research, Curacle Co. Ltd, Seoul, Republic of Korea; ^3^ Brain Korea 21 (BK21) FOUR Program, Yonsei Education and Research Center for Biosystems, Yonsei University, Seoul, Republic of Korea

**Keywords:** CU06-1004, inflammatory bowel disease (IBD), colitis-associated colorectal cancer (CAC), dextran sodium sulfate (DSS), azoxymethane (AOM), tumorigenesis

## Abstract

**Background:**

Ulcerative colitis (UC), a type of inflammatory bowel disease (IBD), is a chronic inflammatory disorder of the colon. Chronic intestinal inflammation plays a critical role in the increased risk of developing colitis-associated cancer (CAC). CU06-1004, an endothelial dysfunction blocker, can alleviate acute colitis by suppressing inflammation and regulating colonic vascular dysfunction. However, whether CU06-1004 suppresses chronic intestinal inflammation and prevents the development of CAC remains unclear.

**Methods:**

In this study, we investigated the protective effects of CU06-1004 by suppressing inflammation in both the dextran sulfate sodium (DSS)-induced chronic colitis model and the azoxymethane (AOM)/DSS-induced colorectal cancer mouse models. We evaluated the expression of key pro-inflammatory cytokines, assessed histological characteristics in the animals, and examined the expression of key genes associated with inflammation.

**Results:**

In the DSS-induced chronic colitis model, our results showed that CU06-1004 treatment suppressed inflammation, as evidenced by disease activity index scores, colon length, colon damage, and histological analysis. Furthermore, CU06-1004 administration reduced the levels of various inflammatory cytokines and factors (tumor necrosis factor-α, interleukin (IL)-1β, IL-6, cyclooxygenase-2, and inducible nitric oxide synthase), decreased immune cell infiltration (F4/80+ macrophages and CD177+ neutrophils), and alleviated inflammation by inhibiting vascular adhesion molecules. Moreover, in the AOM/DSS-induced colorectal cancer model as well, CU06-1004 significantly reduced the severity of colitis. CU06-1004 treatment also significantly reduced both the number and size of AOM/DSS-induced colorectal tumors, suppressed inflammation, and inhibited the malignant proliferation of epithelial cells. Additionally, CU06-1004 treatment downregulated the expression of the key colorectal cancer marker β-catenin and its target gene c-Myc in AOM/DSS-induced mice, thereby inhibiting tumor growth.

**Conclusion:**

Our findings suggest that CU06-1004 inhibits inflammation-induced tumorigenesis by modulating the inflammatory response in the colon. Consequently, CU06-1004 could represent a promising therapeutic candidate for the prevention of colorectal cancer through modulation of inflammation.

## 1 Introduction

Cell proliferation is a fundamental feature of living tissues, but when dysregulated, it can lead to carcinogenesis (Markert et al., 2012). Colorectal cancer (CRC) is the third most commonly diagnosed cancer globally and is strongly linked to chronic inflammation, a key driver of tumor progression (Chumanevich et al., 2010; Hanahan and Weinberg, 2011; Siegel et al., 2020). Inflammatory bowel disease (IBD), including Crohn’s disease and ulcerative colitis, significantly increases the risk of CRC, often progressing to colitis-associated colorectal cancer (CAC) (Cummings and Rubin, 2006; Rieder et al., 2017). This risk escalates with the duration and severity of inflammation, with studies reporting an increase from 0.02% after 10 years to 13.9% after 30 years in patients with ulcerative colitis (Niu et al., 2015; Bopanna et al., 2017). The transition from IBD to CAC involves sustained immune cell infiltration, pro-inflammatory cytokine production, and oxidative stress, which collectively disrupt cellular homeostasis and drive the transformation of normal epithelial cells into dysplastic and metastatic adenocarcinomas (Grivennikov and Karin, 2010; Coskun et al., 2017; Gowrikumar et al., 2019; Vaghari-Tabari et al., 2022). Cytokines, such as tumor necrosis factor-alpha (TNF-α), interleukin (IL)-6, and IL-1, play central roles in mediating these inflammatory responses by promoting immune cell recruitment and amplifying the inflammatory cascade (Vaghari-Tabari et al., 2022). In parallel, cell adhesion molecules (CAMs), including intercellular adhesion molecule (ICAM)-1, vascular cell adhesion molecule (VCAM)-1, and mucosal addressin cell adhesion molecule (MAdCAM)-1, facilitate leukocyte extravasation into inflamed mucosa through multistep processes involving rolling, adhesion, and transmigration, thereby sustaining chronic inflammation in the intestinal microenvironment (Panes and Granger, 1998; Gu et al., 2017). These inflammatory mediators also contribute to tumor-promoting signaling through activation of oncogenic transcription factors such as nuclear factor kappa-light-chain-enhancer of activated B cells (NF-κB) and signal transducer and activator of transcription 3 (STAT3), further accelerating CAC development (Grivennikov et al., 2009; Rahat and Shakya, 2016; Wu et al., 2016). Chronic inflammation is a key driver in the progression of CAC, rendering its effective suppression critical for cancer prevention. Notably, targeting cytokines has been shown to reduce inflammation and tumor burden in preclinical models, suggesting their therapeutic potential in preventing the progression from IBD to CRC (Popivanova et al., 2008; Grivennikov et al., 2009).

Current treatments for IBD, including 5-aminosalicylic acid (5-ASA), corticosteroids, biologics, and immunosuppressants, are designed to alleviate inflammation and related symptoms (Gao et al., 2020). Recently, small molecules have garnered interest as promising therapeutic agents for both IBD and colorectal cancer due to their ability to modulate disease-related molecular pathways (Vaghari-Tabari et al., 2020). However, many of these therapies are associated with an increased risk of infections and various adverse effects (Lautenschlager et al., 2014; Gong et al., 2018). In advanced stages, surgical options such as total colectomy or prophylactic colectomy may be considered; however, these approaches do not completely eliminate the risk of cancer. Furthermore, chemotherapy is often constrained by toxicity and poor patient tolerability (Mishra and Chaurasia, 2017; Wang et al., 2019). These limitations highlight the urgent need for safer and more effective strategies to prevent the progression from IBD to CRC.

CU06-1004 is an effective agent for blocking endothelial dysfunction by activating the cAMP/Rac/cortactin signaling pathway, which promotes cortical actin ring formation, thereby reducing vascular hyperpermeability and enhancing the survival and integrity of endothelial cells (Maharjan et al., 2013; Zhang et al., 2017). It prevents the loss of junction proteins and vascular leakage induced by inflammatory stimuli, vascular endothelial growth factor (VEGF), and histamine (Maharjan et al., 2013; Zhang et al., 2017). Furthermore, CU06-1004 suppresses the expression of inflammatory adhesion molecules, such as ICAM-1 and VCAM-1, by inhibiting NF-κB signaling, thereby contributing to vascular stability and endothelial barrier reinforcement (Park et al., 2020; Kim et al., 2023). In support of its vascular-focused action, CU06-1004 has also been shown to alleviate BK-induced vascular hyperpermeability in a murine hereditary angioedema model by protecting endothelial junction integrity (Lee et al., 2023), and to enhance vascular integrity and reduce edema and inflammation in myocardial ischemia–reperfusion injury by preserving junction proteins and reducing VCAM-1 expression (Zhang et al., 2022). Its therapeutic efficacy has been demonstrated across various disease models, including inflammation, cancer, stroke, and diabetic retinopathy (Kim D. Y. et al., 2020; Kim Y. S. et al., 2020; Park et al., 2020; Zhang et al., 2022; Kim et al., 2023; Noh et al., 2023). Notably, our previous study demonstrated that CU06-1004 alleviates colonic vascular dysfunction and attenuates inflammation in a DSS-induced acute colitis model (Kim Y. S. et al., 2020). Specifically, CU06-1004 treatment restored vascular architecture, improved clinical disease activity scores, and enhanced the expression of tight junction proteins, thereby strengthening endothelial barrier integrity and reducing mucosal injury. While these findings highlight its therapeutic potential in acute inflammation, the chronic and progressive characteristics of colitis-associated cancer (CAC) warrant further investigation into the long-term effects of CU06-1004. To date, the efficacy and underlying mechanisms of CU06-1004 in models of chronic intestinal inflammation and CAC have not been fully elucidated. Elucidating these aspects is essential for validating CU06-1004 as a novel chemopreventive agent targeting vascular dysfunction in inflammation-driven colorectal carcinogenesis.

The DSS-induced colitis model mimics human ulcerative colitis by inducing both acute and chronic intestinal inflammation, making it a widely used and reliable model for IBD research (Poritz et al., 2007; Yan et al., 2009; Chassaing et al., 2014). The AOM/DSS model recapitulates inflammation-driven colorectal tumorigenesis by combining chemical carcinogenesis with chronic colitis, enabling reproducible tumor development within 10 weeks (Tanaka et al., 2003; Chen and Huang, 2009; De Robertis et al., 2011; Parang et al., 2016). In this study, we aimed to utilize two well-established murine models: the Dextran Sulfate Sodium (DSS)-induced chronic colitis model and the Azoxymethane (AOM)/DSS-induced CAC model. These models allowed us to assess the therapeutic potential of CU06-1004 under conditions that more closely resemble the chronic inflammatory environment observed in human disease. By evaluating histological and molecular markers associated with inflammation, vascular dysfunction, and carcinogenesis, we aimed to determine whether CU06-1004 can suppress chronic colonic inflammation and prevent the development of inflammation-driven colorectal tumors. Together, these findings aim to clarify the long-term therapeutic potential of CU06-1004 in preventing chronic inflammation-driven colorectal tumorigenesis, thereby offering a promising strategy for the management of CAC.

## 2 Materials and methods

### 2.1 Chemical and drugs

CU06-1004 was synthesized as previously described (Maharjan et al., 2013). Briefly, CU06-1004 was synthesized via tetrahydropyran deprotection and subsequent glycosidation with 4,6-di-O-acetyl-2,3-didieoxyhex-2-enopyran in the presence of an acid. DSS (36,000–50,000 Da, 0216011090) was purchased from MP Biochemicals (Solon, OH, USA), and AOM was ordered from Sigma-Aldrich.

### 2.2 Mice

Male ICR mice (aged 4–5 weeks, weighing 26–28 g) were purchased from DBL (Eumseong, Chungcheongbuk-do, Republic of Korea). All mice were housed under controlled, specific pathogen–free conditions (12-h/12-h dark-light cycle, 22 °C ± 1 °C, 50%–60% humidity) and allowed *ad libitum* access to a commercial diet and tap water. All animal experiments were approved in advance by the Animal Care and Use Committee of Yonsei University (Seoul, Republic of Korea) and conducted in accordance with established guidelines (IACUC-A-202011-1163-01).

### 2.3 DSS-induced chronic colitis model

To the DSS-induced chronic colitis model, mice were acclimated for 1 week and randomly assigned to three groups: Control, DSS, and CU06-1004 treatment. Chronic colitis was induced by administering 2.5% DSS in drinking water for three cycles, each consisting of 1 week of DSS treatment followed by 2 weeks of regular water. Starting on the first day of DSS administration, mice in the CU06-1004 group received a daily oral gavage of CU06-1004 (10 mg/kg), dissolved in olive oil and administered in a volume of 100 μL, for 9 consecutive weeks. Control and DSS groups received an equivalent volume of olive oil as a vehicle control. The dosage of CU06-1004 was determined based on previous dose–response studies conducted in our laboratory (Kim Y. S. et al., 2020).

### 2.4 AOM/DSS-induced CAC model

For this model, mice were acclimated for 1 week and then randomly assigned to three groups: Control, AOM/DSS, and CU06-1004 treatment groups (10 mg/kg). Mice in the AOM/DSS and CU06-1004 groups received an intraperitoneal injection of AOM (10 mg/kg), followed by three cycles involving administration of 2.5% DSS in drinking water (Weeks 1–2, 4–5, and 7–8). From the first day of AOM injection, CU06-1004 was administered daily via oral gavage at doses of 10 mg/kg for 10 weeks. Control and AOM/DSS groups received an equivalent volume of olive oil as a vehicle control throughout the experimental period.

### 2.5 Clinical assessment of DSS-induced chronic colitis and AOM/DSS-induced colorectal cancer

Mice were examined twice a week for body weight, stool consistency, and the presence of fecal blood. The Disease Activity Index (DAI) was determined as previously described (Cooper et al., 1993): (1) weight loss (0 = <1%, 1 = 1%–5%, 2 = 5%–10%, 3 = 10%–20%, 4 = >20%); (2) stool consistency (0 = normal, 2 = loose stool, 4 = diarrhea); and (3) fecal blood (0 = no blood, 2 = red, 4 = black) (Supplementary Table S1). Weight loss was calculated based on the weight recorded on day 0 for each mouse. Colitis severity was assessed by evaluating these clinical disease activities. The DAI score was calculated as the sum of these scores, ranging from 0 (healthy) to 12 (severe colitis).

### 2.6 Collection of tissue and serum samples

At the end of the experiment, all mice were deeply anesthetized with Avertin (2,2,2-tribromoethanol; 240 mg/kg, intraperitoneally; Sigma-Aldrich, USA). After confirming the absence of reflexes, euthanasia was performed by carbon dioxide (CO_2_) inhalation. CO_2_ gas was introduced into the chamber without pre-charging, at a fill rate of 50% of the chamber volume per minute (5 L/min for a 10 L chamber). After measuring the weight and length of the colon, tumors were subjected to quantitative analysis. The distal part of the colon was excised to collect tissue for RNA analysis, and blood samples were obtained via cardiac puncture for serum collection. The tissues were fixed in 4% paraformaldehyde for histological analysis.

### 2.7 Histopathological analysis

After fixation for 48 h with 4% paraformaldehyde, the formalin-fixed colon samples were embedded in paraffin wax. Sections of 5-μm thickness were cut using a microtome (Leica, Wetzlar, Germany) and stained with hematoxylin and eosin (H&E) for histological analysis. The stained samples were examined under an optical microscope (Nikon, Tokyo, Japan), and the images were graded on a scale from 0 to 4. Epithelial loss, crypt disruption, and inflammatory cell infiltration were graded separately as follows: (0 = no change, 1 = localized and mild, 2 = localized and moderate, 3 = extensive and moderate, and 4 = extensive and severe). The three grades were then combined to calculate a total histological score. Tissue pathology was scored using previously described systems (Kim Y. S. et al., 2020).

### 2.8 Immunohistochemistry analysis

For immunohistochemistry staining, each tissue section was de-paraffinized and rehydrated using a graded series of ethanol (100%, 90%, 80%, 70%, and 50%) followed by treatment with distilled water. Antigen retrieval was performed by boiling the slides in citrate buffer (10 mM sodium citrate, 0.05% Tween 20, pH 6.0) in a microwave oven for 10 min, followed by cooling at room temperature. The slides were then incubated with 0.04% Triton X-100 in PBS for 5 min, followed by treatment with 3% H_2_O_2_ in methanol for 10 min. After washing with 0.1% Tween 20 in PBS, the sections were blocked with Protein Block solution (Dako, Santa Cruz, CA, USA) for 1 h. After washing, the tissues were incubated with the following primary antibodies overnight at 4 °C: F4/80(MF48000), CD177(sc-374291), Ki67(550609), β-catenin(sc-7963), and c-Myc (ab32072). The sections were then washed thrice in 0.1% Tween 20 in PBS and incubated with secondary antibodies. After washing, the sections were developed with DAB using a commercial kit (Dako, K5007) and counterstained with hematoxylin. Finally, observations and photographs were taken using an optical microscope (Nikon, Tokyo, Japan).

### 2.9 Quantification of cytokine levels in serum using ELISA

Blood was collected in a serum-separating tube (Becton Dickinson, 365967) and incubated at room temperature for 30 min. The samples were then centrifuged at 3,000 *g* for 10 min at room temperature to obtain murine serum. The serum concentrations of TNF-α, IL-6, and IL-1β were measured using the Quantikine ELISA Kit (R&D Systems, MTA00B, M6000B, and MLB00C) following the manufacturer’s protocol.

### 2.10 Colon RNA extraction and real-time polymerase chain reaction

Total RNA was extracted from colon tissues using easy-BLUE™ (Intron, Seongnam, Korea) following the manufacturer’s instructions. After extraction, RNA was purified using lithium chloride and subjected to sodium acetate precipitation to remove any residual DSS, a potential polymerase inhibitor. Subsequently, cDNA was synthesized from the purified RNA using M-MLV Reverse Transcriptase (Promega, Madison, WI, USA) in the presence of oligo(dT) primers and dNTP. Quantitative real-time polymerase chain reaction (qRT-PCR) was performed using a SYBR Green master mix (Thermo Scientific, Waltham, MA, USA). Primer sequences are listed in Supplementary Table S2. The expression levels were calculated using the 2^−ΔΔCT^ method, and the Ct values were normalized using *GAPDH* as the reference gene.

### 2.11 Statistical analysis

Analysis of variance (ANOVA) was used for comparisons between groups. The differences in means among groups were statistically analyzed using one-way or two-way ANOVA, followed by Tukey’s test. Data are presented as the mean ± standard deviation (SD) or mean ± standard error of the mean (SEM). A p-value of less than 0.05 was considered significant. Kaplan–Meier survival curves were compared using the log-rank test, with a *P* value <0.05 considered statistically significant. All statistical analyses were performed using GraphPad Prism version 8 (GraphPad Software, Inc., La Jolla, CA, USA).

## 3 Results

### 3.1 CU06-1004 mitigates the progression of DSS-induced colonic inflammation in a chronic colitis mouse model

**FIGURE 1:**
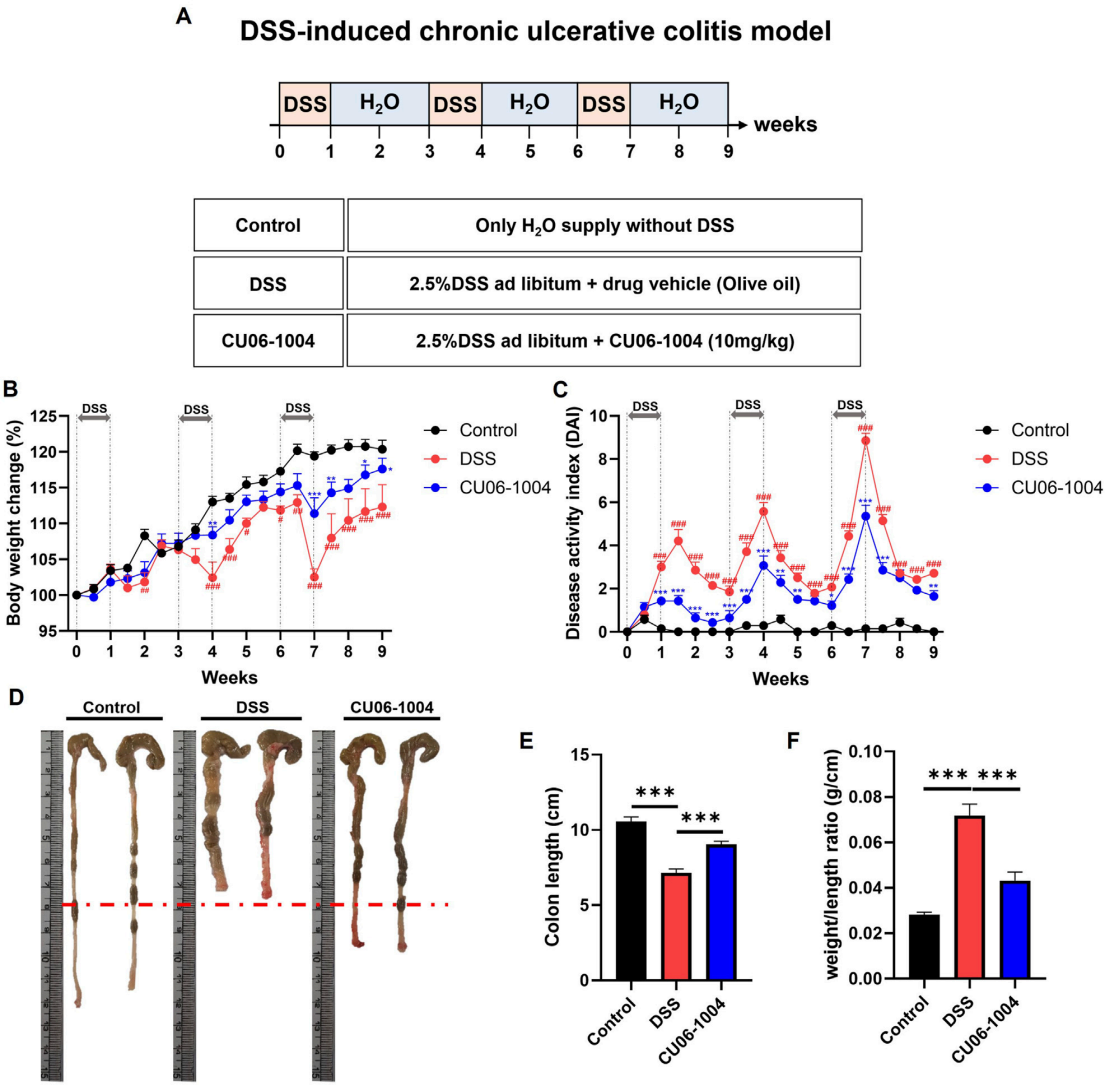
Effect of CU06-1004 on the severity of dextran sulfate sodium (DSS)-induced chronic colitis in mice. **(A)** Schematic diagram depicting the development of the DSS-induced chronic colitis mouse model and CU06-1004 administration. **(B)** Body weight changes were calculated as a percentage of the weight recorded on day 0. n = 7 per group. **(C)** Disease Activity Index (DAI) score throughout the experimental period. n = 7 per group. Statistical significance in **(B,C)** is indicated as follows: *P < 0.05, **P < 0.01, ***P < 0.001 vs. DSS group; ^#^ *P* < 0.05, ^##^ *P* < 0.01, ^###^ *P* < 0.001 vs. Control group. **(D)** Macroscopic view of a representative whole colon. **(E)** Colon length contraction, which serves as an indirect marker of inflammation, was measured at the time of euthanasia. n = 8 per group. **(F)** Colon weight-to-length ratio. n = 8 per group. Data presented as mean ± SEM values; *P < 0.05; **P < 0.01; ***P < 0.001.

To investigate whether CU06-1004 could prevent the progression of DSS-induced colonic inflammation into chronic colitis, we employed a chronic colitis mouse model by administering 2.5% DSS in drinking water for 1 week, followed by 2 weeks of regular water, repeated for a total of three cycles (Figure 1A). Repeated DSS exposure led to progressive bodyweight loss, whereas CU06-1004 treatment significantly alleviated this decline (Figure 1B). The DAI, which includes parameters such as weight loss, diarrhea, and rectal bleeding, was significantly lower in the CU06-1004-treated group compared to that in the DSS group (Figure 1C). Additionally, DSS treatment led to marked colon shortening—an indicator of chronic inflammation (Salvo et al., 2020)—which was significantly prevented by CU06-1004 treatment (Figures 1D,E). The colon weight-to-length ratio, a measure of edema and tissue inflammation, was also significantly reduced in the CU06-1004 group (Figure 1F). Furthermore, DSS-induced splenomegaly, reflecting systemic immune activation, was markedly attenuated by CU06-1004 (Supplementary Figure S1A). Taken together, these results suggest that CU06-1004 effectively inhibited the progression of DSS-induced colonic inflammation toward a chronic colitic state.

### 3.2 CU06-1004 inhibits histological damage and immune cell infiltration during chronic colitis progression in DSS-treated mice

**FIGURE 2:**
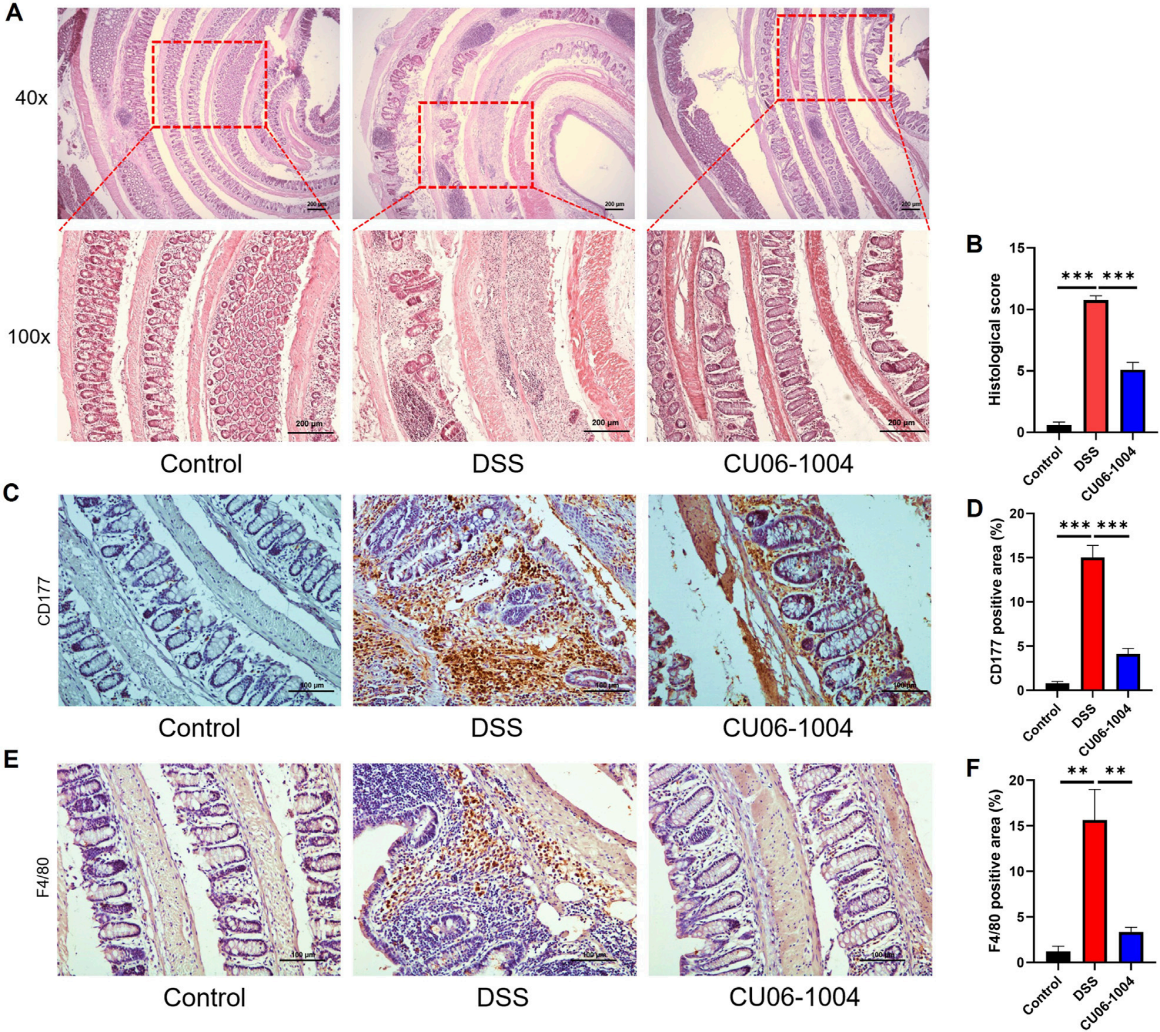
CU06-1004 suppressed histopathological damage and immune cell infiltration in DSS-induced chronic colitis mouse models. **(A)** Colon sections were stained with hematoxylin and eosin (H%E). (×40 magnification, upper panels; ×100 magnification, lower panels; Scale bar: 200 μm). **(B)** Histopathologic injury score was significantly reduced in the CU06-1004 group after induction of colitis following DSS administration. n = 5–9 per group. **(C)** Representative IHC staining images for CD177 in the distal colon. (Scale bar: 100 μm). **(D)** The average percentage of CD177-positive cells in the distal colon. The number of positive cells was counted in the inflamed mucosa. n = 5–9 per group. **(E)** Representative IHC staining images of F4/80 in the distal colon. (Scale bar: 100 μm). **(F)** The average percentage of F4/80-positive cells in the distal colon. The number of positive cells was counted in the inflamed mucosa. n = 5–9 per group. Data presented as mean ± SEM values; **P < 0.01; ***P < 0.001.

To further evaluate the protective effect of CU06-1004 against the progression of chronic colitis, we performed histopathological and immunohistochemical analyses of colon tissues. H&E staining of the colon sections harvested from DSS-treated mice revealed severe epithelial disruption, loss of crypt structures, widespread mucosal edema, and massive infiltration of inflammatory cells, features characteristic of chronic intestinal injury (Xu et al., 2020). Notably, CU06-1004 treatment markedly preserved tissue architecture and attenuated these pathological changes (Figure 2A). Histological scores reflecting epithelial cell loss, crypt damage, and immune cell infiltration were significantly reduced in the CU06-1004 group compared to those in the DSS group, supporting the histological findings (Figure 2B). Given the role of immune cell recruitment in perpetuating chronic inflammation, we next examined the infiltration of neutrophils and macrophages. IHC analysis revealed a marked increase in CD177^+^ neutrophils in the colonic mucosa of DSS-treated mice. However, CU06-1004 significantly reduced this DSS-induced neutrophil infiltration (Figures 2C,D). Similarly, the number of F4/80^+^ macrophages, which accumulate following neutrophil infiltration (Jones et al., 2018), was also significantly decreased in the CU06-1004 group compared to the DSS group (Figures 2E,F). These findings collectively suggest that CU06-1004 not only preserved colonic tissue integrity but also prevented the sustained recruitment of inflammatory immune cells, thereby impeding the progression of chronic intestinal inflammation.

### 3.3 CU06-1004 suppresses inflammatory mediators in colon tissue and serum, preventing the progression of inflammation in DSS-induced chronic colitis

**FIGURE 3:**
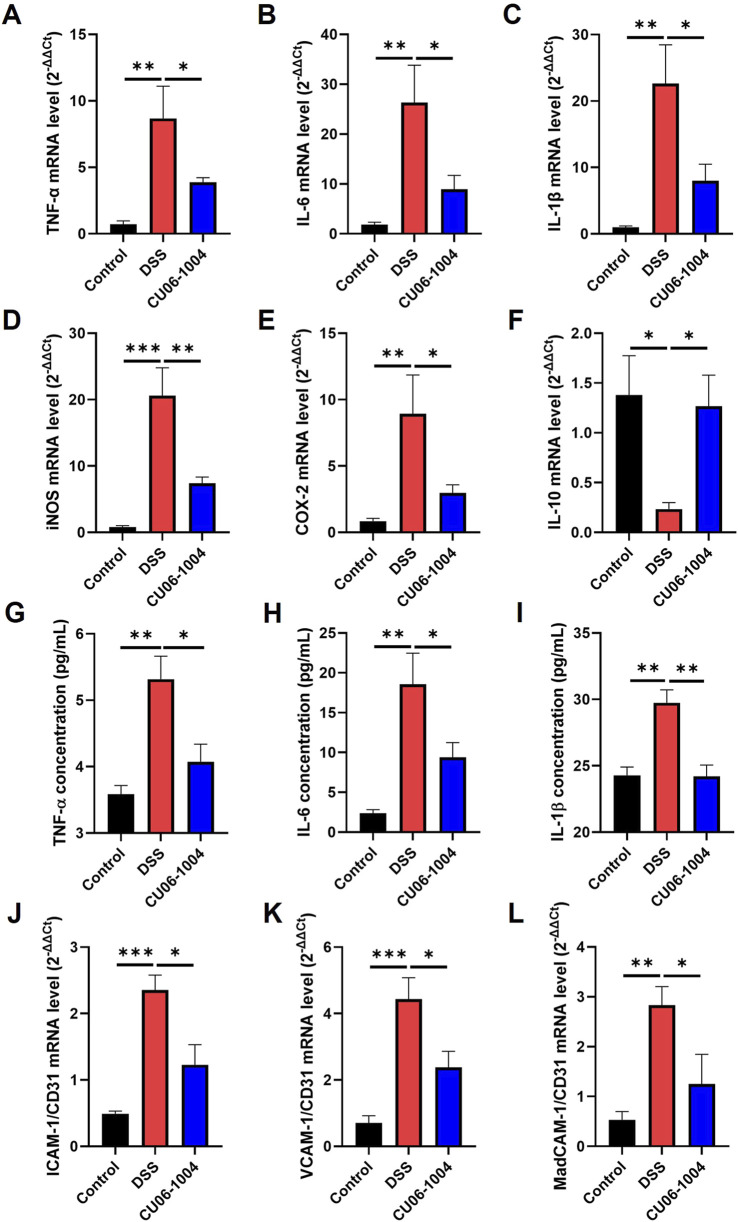
CU06-1004 attenuated the expression levels of inflammation-related factors and vascular adhesion molecules in a dextran sulfate sodium (DSS)-induced chronic colitis mouse model. **(A–C)** mRNA expression levels of pro-inflammatory cytokines (*TNF-*α*, IL-6*, and *IL-1*β) in the colon tissue were measured using reverse transcriptase–polymerase chain reaction. n = 4–6 per group. **(D–F)** mRNA expression levels of inflammation-related genes (*iNOS, COX-2*, and *IL-10*) in the colon tissue were measured using reverse transcriptase–polymerase chain reaction. n = 4–6 per group. **(G–I)** The serum concentration of inflammatory cytokines (TNF-α, IL-6, and IL-1β) was measured using ELISA. n = 4–7 per group. **(J–L)** mRNA expression levels of vascular adhesion molecules (*ICAM-1, VCAM-1,* and *MAdCAM-1*) in the colon tissue were measured using reverse transcriptase–polymerase chain reaction. Adhesion molecules represent the relative ratio of *CD31* mRNA expression. n = 4–6 per group. TNF-α, tumor necrosis factor-alpha; IL-6, interleukin-6; IL-1β, Interleukin-1β; IL-10, interleukin-10; COX-2, cyclooxygenase-2; iNOS, inducible nitric oxide synthase; ICAM-1, intracellular adhesion molecule 1; VCAM-1, vascular cell adhesion molecule 1; MAdCAM-1, mucosal addressin cell adhesion molecule 1; Data presented as mean ± SEM values; *P < 0.05; **P < 0.01; ***P < 0.001.

To explore the molecular mechanisms underlying the protective effects of CU06-1004 against chronic intestinal inflammation, we analyzed the expression of inflammation-related cytokines and enzymes in colon tissue and serum. DSS administration markedly upregulated the mRNA levels of genes encoding pro-inflammatory cytokines, including *TNF-*α*, IL-6*, and *IL-1*β, in the colon compared to those recorded in the control group, indicating the progression toward chronic inflammation. However, CU06-1004 treatment significantly attenuated this increase (Figures 3A–C). In addition, the expression of inflammatory enzymes inducible nitric oxide synthase (iNOS) and cyclooxygenase-2 (COX-2), which are commonly elevated in inflamed colonic tissue (Kohno et al., 2005; Krieglstein et al., 2007), was also significantly reduced in the CU06-1004 group (Figures 3D,E). Conversely, the mRNA expression of *IL-10*, a representative anti-inflammatory cytokine that was significantly suppressed in the DSS group, was preserved in mice treated with CU06-1004 (Figure 3F). Beyond local inflammation, systemic inflammatory markers were also assessed. ELISA measurements of serum TNF-α, IL-6, and IL-1β levels showed significant elevation in DSS-treated mice. CU06-1004 administration effectively reversed these increases, further supporting its anti-inflammatory activity at the systemic level (Figures 3G–I). Taken together, these results suggest that CU06-1004 hinders the escalation of mucosal and systemic inflammation by modulating key pro- and anti-inflammatory mediators, thereby potentially preventing the progression to chronic colitis.

### 3.4 CU06-1004 inhibits the expression of vascular adhesion molecules to prevent leukocyte recruitment in DSS-induced chronic colitis

To determine whether CU06-1004 interferes with this recruitment process, we examined the expression of endothelial adhesion molecules in the colons of DSS-treated mice. We measured the expression of adhesion molecule genes specifically in the blood vessels of the colon tissue exhibiting chronic inflammation. The mRNA expression of *ICAM-1, VCAM-1,* and *MAdCAM-1*, which are key mediators of leukocyte adhesion and transmigration (Gao et al., 2021; Singh et al., 2023), was significantly elevated in the DSS group compared to that in the control group, as determined by normalization to the vascular marker CD31. Notably, CU06-1004 treatment markedly suppressed the upregulation of all three adhesion molecules (Figures 3J–L). Given that these adhesion molecules facilitate the entry of neutrophils and macrophages into inflamed tissues, their suppression by CU06-1004 likely contributed to the observed reduction in immune cell infiltration. These findings suggest that CU06-1004 protects against chronic intestinal inflammation, in part, by limiting leukocyte recruitment through the inhibition of vascular adhesion molecule expression.

### 3.5 CU06-1004 suppresses the progression of colonic inflammation in an AOM/DSS-induced mouse model of colorectal cancer

**FIGURE 4:**
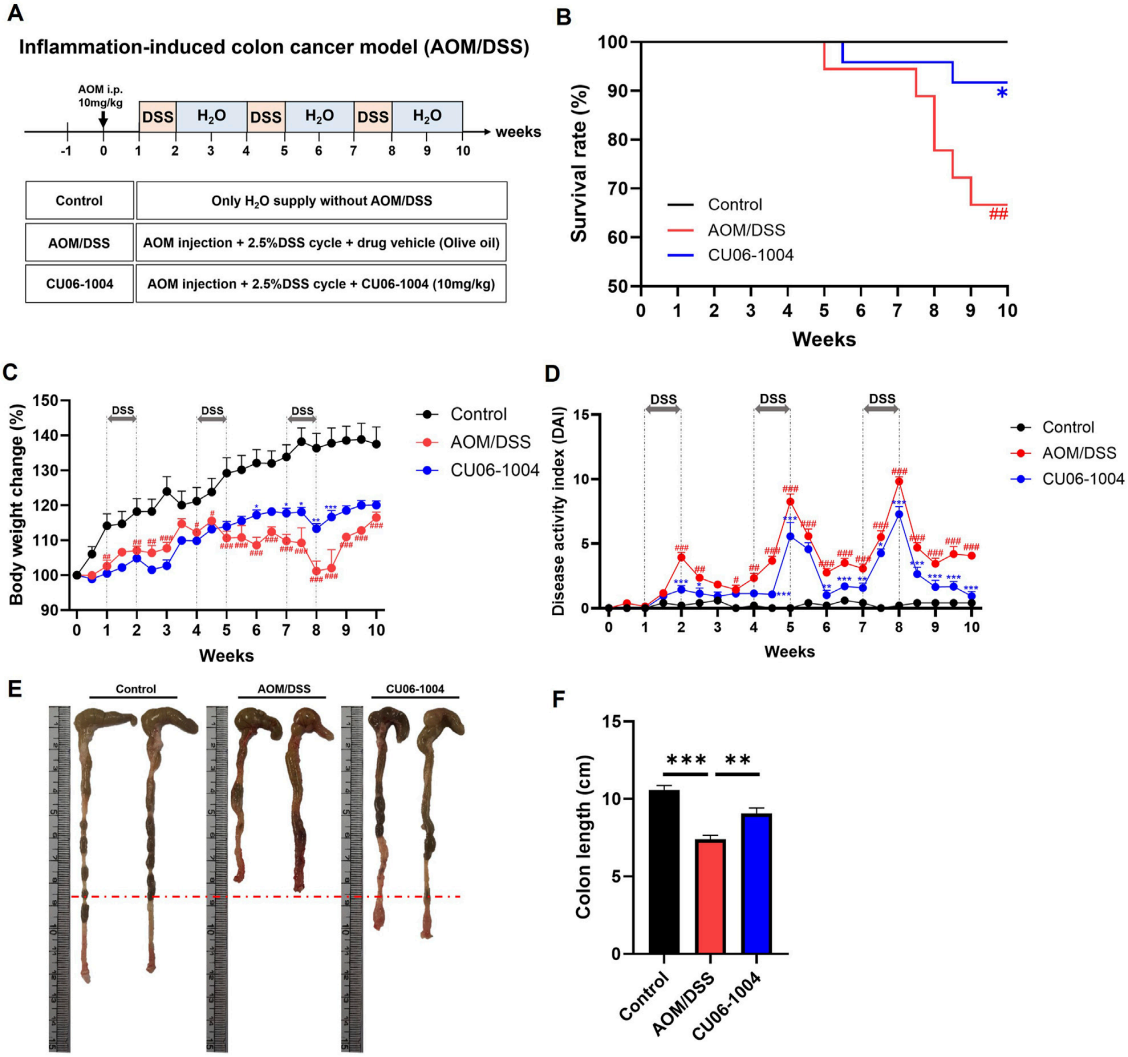
Effect of CU06-1004 on the severity of Azoxymethane (AOM)/DSS-induced colorectal cancer in mice. **(A)** Schematic diagram of the development of AOM/DSS-induced colorectal cancer mouse model and CU06-1004 administration. **(B)** Survival rates (n = 18–24 per group) revealed that CU06-1004 significantly extended survival in mice, as determined by log-rank tests. Statistical significance is indicated as follows: *P < 0.05 vs. AOM/DSS group; ^##^ *P* < 0.01 vs. Control group. **(C)** Body weight changes were calculated as a percentage of the weight recorded on day 0. n = 6–8 per group. **(D)** Disease Activity Index (DAI) score throughout the experimental period. n = 5–9 per group. Statistical significance in **(C,D)** is indicated as follows: *P < 0.05, **P < 0.01, ***P < 0.001 vs. AOM/DSS group; ^#^ *P* < 0.05, ^##^ *P* < 0.01, ^###^ *P* < 0.001 vs. Control group. **(E)** Macroscopic view of a representative whole colon. **(F)** Colon length contraction serves as an indirect marker of inflammation and was measured at the time of euthanasia. n = 7–8 per group. Data presented as mean ± SEM values; **P < 0.01; ***P < 0.001.

To investigate whether CU06-1004 can suppress the progression from chronic inflammation to colorectal cancer, we employed the AOM/DSS-induced colitis-associated CAC mouse model. Mice received an intraperitoneal injection of AOM (10 mg/kg), followed by three cycles of 2.5% DSS for 1 week and regular water for 2 weeks, mimicking chronic colitis-driven tumorigenesis (Figure 4A). AOM/DSS treatment induced severe symptoms, including body weight loss, increased mortality, and rectal bleeding. Notably, CU06-1004 treatment significantly prolonged survival time (Figure 4B) and mitigated body weight loss over the course of the model (Figure 4C). The DAI, measured by weight loss, diarrhea, and rectal bleeding, also showed marked improvement in the CU06-1004 group compared to the AOM/DSS group (Figure 4D). Additionally, CU06-1004 alleviated colon shortening, a hallmark of severe colonic inflammation (Figures 4E,F). Spleen weights, which typically increased in response to systemic inflammation (Hanscom et al., 2021), were significantly reduced in the CU06-1004 group compared to those in the untreated AOM/DSS group (Supplementary Figure S1B). Notably, rectal prolapse, a symptom commonly observed in advanced inflammation-associated CRC (Zheng et al., 2016), was prominent in AOM/DSS mice but was notably absent in CU06-1004-treated mice (Supplementary Figure S2). These findings suggest that CU06-1004 effectively suppresses the severity of inflammation and early tumor-promoting conditions in a mouse model of inflammation-associated colorectal cancer.

### 3.6 CU06-1004 suppresses tumor development, immune cells infiltration, and inflammation in AOM/DSS-induced colitis-associated colorectal cancer mouse models

**FIGURE 5:**
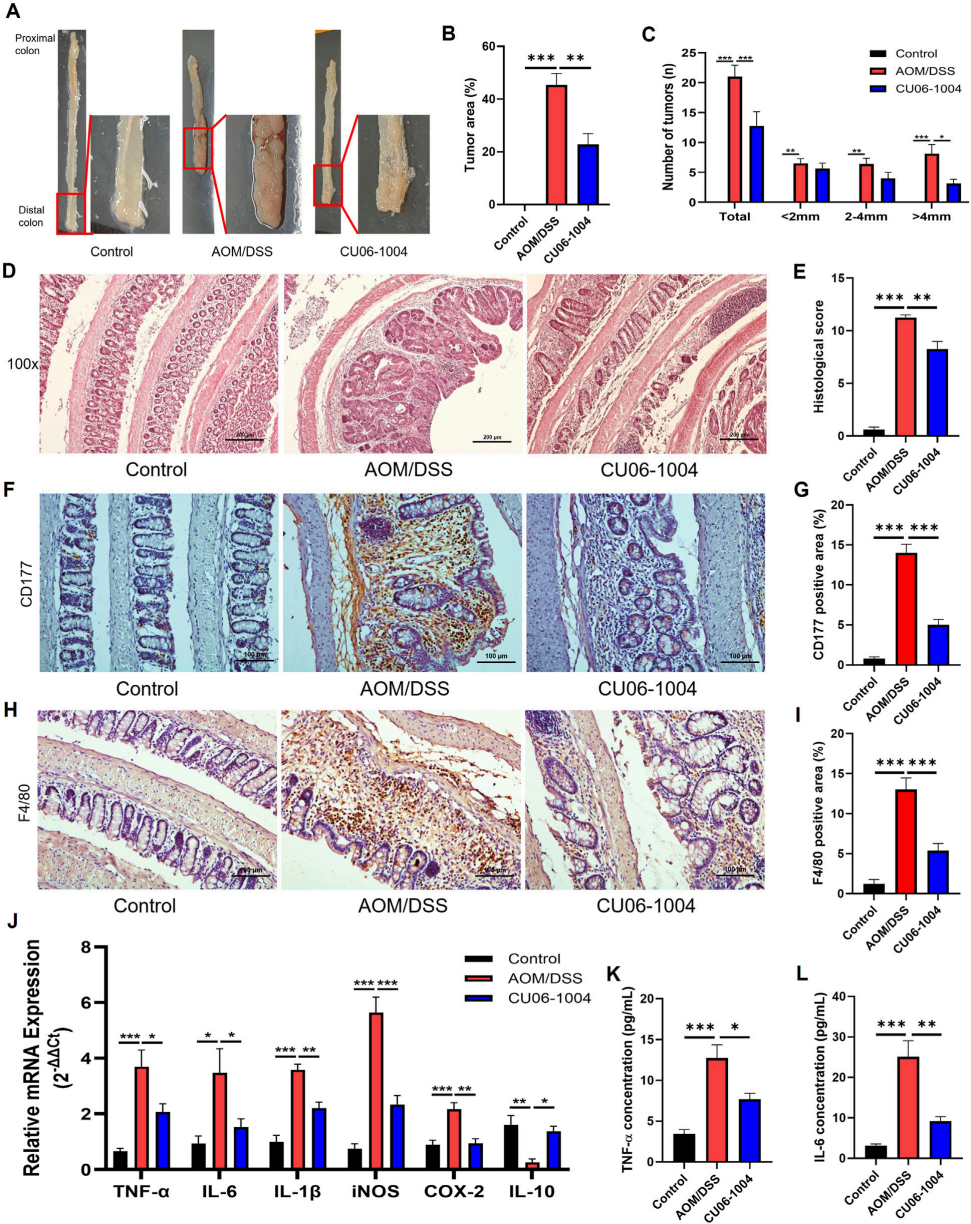
CU06-1004 attenuates tumor progression and inflammation in AOM/DSS-induced colorectal cancer mouse models. **(A)** Macroscopic view of a representative lumen of the colon. **(B)** The percentage of tumor area in the colon. n = 5-8 per group. **(C)** Number and size of tumors in each mouse. CU06-1004 significantly reduced the total tumor count and inhibited tumor growth. n = 5-8 per group. **(D)** Colon sections were stained with H&E. (Scale bar: 200 μm). **(E)** Histopathologic injury score was significantly reduced in the CU06-1004 group after colorectal cancer induction following AOM/DSS treatment. n = 5–8 per group. **(F,H)** Representative IHC images of CD177^+^ neutrophils **(F)** and F4/80^+^ macrophages **(H)** in the inflamed distal colonic mucosa. Images were acquired at ×200 magnification (scale bar: 100 μm). **(G,I)** Quantification of CD177^+^ **(G)** and F4/80^+^ **(I)** cells in inflamed regions. Positive cells were counted, and data are presented as mean percentages per group (n = 5–8). **(J)** mRNA expression levels of pro-inflammatory cytokines (*TNF-*α*, IL-6*, and *IL-1*β), inflammatory enzymes (*iNOS* and *COX-2*), and the anti-inflammatory cytokine (*IL-10)* were measured in colon tissue using reverse transcriptase–polymerase chain reaction. n = 4–6 per group. **(K,L)** The serum concentration of inflammatory cytokine (TNF-α and IL-6) using ELISA. n = 5–8 per group. Data presented as mean ± SEM values; *P < 0.05; **P < 0.01; ***P < 0.001.

To further assess the therapeutic potential of CU06-1004 in CAC, we employed an AOM/DSS-induced CAC mouse model, which reliably develops distal colonic adenomas with 100% incidence (De Robertis et al., 2011). Macroscopic examination revealed that all mice in the AOM/DSS group developed multiple adenomas and hemorrhagic lesions, whereas the CU06-1004-treated group exhibited significantly reduced tumor burden (Figure 5A). Quantitative analysis confirmed that CU06-1004 treatment led to a substantial reduction in tumor area (Figure 5B). While the number of small (<2 mm) and medium-sized (2–4 mm) tumors remained comparable between the AOM/DSS and CU06-1004 groups, the number of large tumors (>4 mm) and the total tumor count per mouse were markedly decreased in the CU06-1004 group (Figure 5C).

Histopathological analysis further supported these observations. H&E staining of Swiss-rolled colon tissue from AOM/DSS-induced mice showed a large polyp in the distal colon, surrounded by inflammatory infiltrate (Parang et al., 2016). Consistently, H&E-stained colon sections from AOM/DSS mice exhibited severe architectural disruption, high-grade epithelial dysplasia, and intense inflammatory infiltration, all of which are hallmarks of advanced adenocarcinoma. In contrast, CU06-1004 preserved the integrity of the intestinal epithelium and reduced mucosal damage (Figure 5D). Quantification of epithelial loss, crypt distortion, and immune cell infiltration in the non-tumor areas confirmed significant histological improvement in the CU06-1004 group (Figure 5E). Therefore, we evaluated the impact of CU06-1004 on immune cell infiltration in colonic tissues using IHC analysis with antibodies to elucidate the immune mechanisms underlying this protective effect. IHC analysis of inflamed colonic mucosa revealed a marked increase in the infiltration of CD177^+^ neutrophils and F4/80^+^ macrophages in the AOM/DSS group, indicating elevated inflammatory activity. In contrast, CU06-1004 treatment significantly reduced the infiltration of these immune cells in the inflamed areas, suggesting an attenuation of inflammatory cell recruitment (Figures 5F–I).

Based on these observations, we further evaluated the expression of inflammation-related molecules contributing to tumorigenesis in the inflamed colon. The mRNA levels of genes encoding key pro-inflammatory cytokines, which include *TNF-*α*, IL-6*, and *IL-1*β, were significantly elevated in colon tissues from the AOM/DSS group but notably suppressed in the CU06-1004 treatment group. Similarly, those of inflammation-associated enzymes *iNOS* and *COX-2* were downregulated upon CU06-1004 administration. Notably, the reduced expression of the anti-inflammatory cytokine IL-10 observed in AOM/DSS mice was restored in the CU06-1004 group (Figure 5J). ELISA analysis of serum samples showed systemic TNF-α and IL-6 levels mirrored tissue findings, with CU06-1004 significantly reducing the levels of these cytokines compared to those in the untreated AOM/DSS group (Figures 5K,L). Taken together, these data demonstrate that CU06-1004 suppresses inflammation-driven tumor formation in the colon by inhibiting immune cell infiltration and production of pro-inflammatory mediators.

### 3.7 CU06-1004 suppresses tumor cell proliferation and oncogenic proteins in the AOM/DSS-induced colitis-associated colorectal cancer model

**FIGURE 6:**
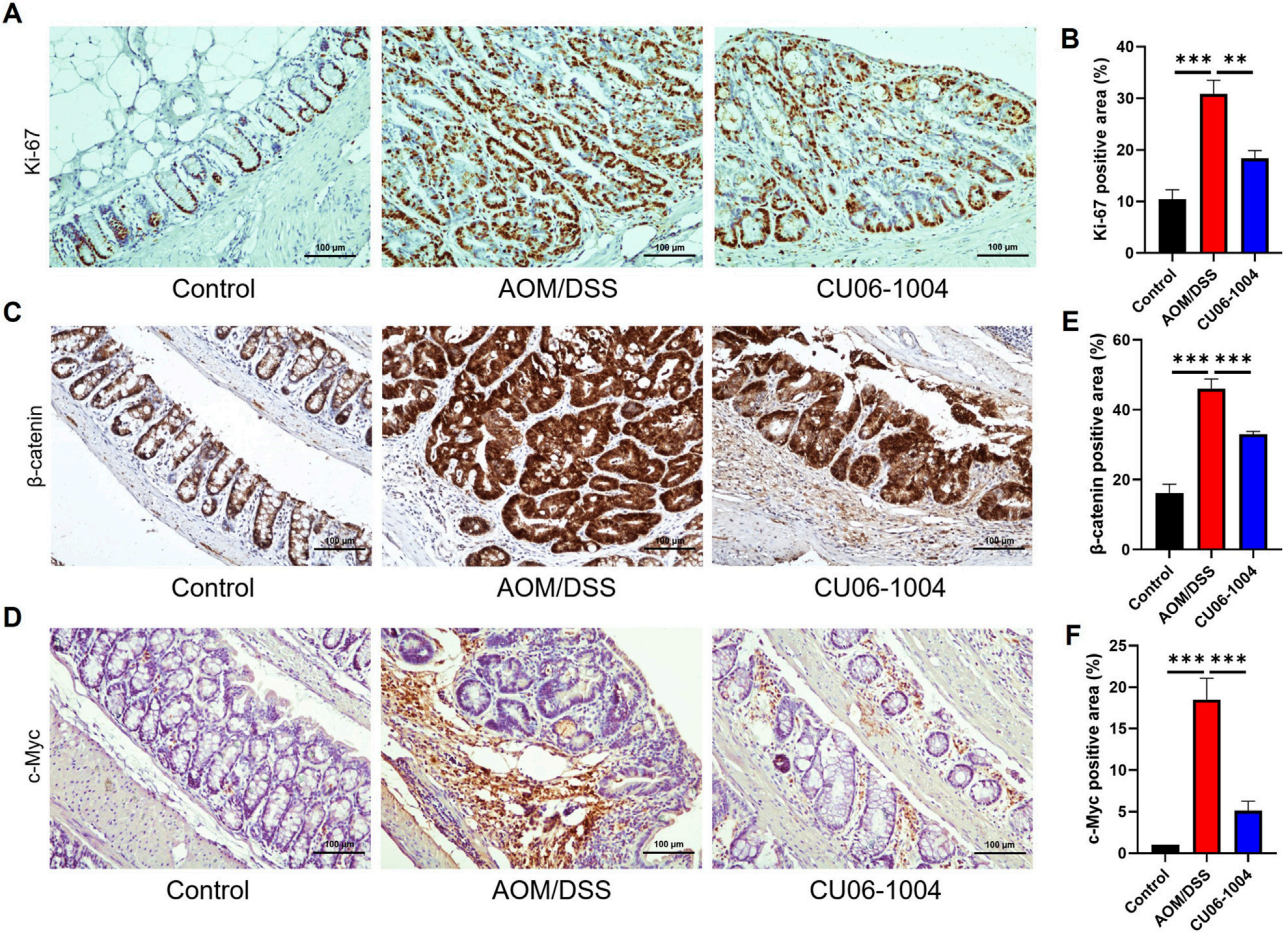
CU06-1004 suppressed the proliferation of colon cells and key proteins associated with colon cancer in AOM/DSS-induced colorectal cancer mouse models. **(A)** Representative IHC staining images for Ki-67 in the distal colon. (Scale bar: 100 μm). Proliferation was assessed by Ki-67 IHC staining. **(B)** The average percentage of Ki-67-positive cells in the distal colon. The number of positive cells in the intestinal crypt was quantified. n = 5–8 per group. **(C)** Representative IHC staining images for β-catenin in the distal colon. (Scale bar: 100 μm). **(D)** Representative IHC staining images for c-Myc in the distal colon. (Scale bar: 100 μm). **(E)** The average percentage of β-catenin-positive cells in the distal colon. The number of positive cells in the intestinal epithelial layer was quantified. n = 5–8 per group. **(F)** The average percentage of c-Myc-positive cells in the distal colon. The number of positive cells in the intestinal epithelial layer was quantified. n = 5–8 per group. c-Myc, cellular myelocytomatosis oncogene; Data presented as mean ± SEM values; **P < 0.01; ***P < 0.001.

To evaluate whether CU06-1004 directly affects tumor cell proliferation in CAC, we performed IHC staining for Ki-67, a well-established marker of cellular proliferation and tumor growth (Hu et al., 2010). The number of Ki-67-positive cells was markedly increased in the colonic epithelium of the AOM/DSS group, indicating active tumor cell proliferation. However, CU06-1004 treatment significantly reduced the number of proliferating cells in the intestinal crypts (Figures 6A,B), suggesting a significant suppression of cell proliferation.

To further explore the molecular mechanisms underlying this suppression, we evaluated components of the Wnt/β-catenin signaling pathway, which plays a critical role in colorectal carcinogenesis (Sebio et al., 2014). We employed IHC analysis to confirm the expression of β-catenin and its downstream protein c-Myc in colon tissue. IHC staining revealed increased accumulation of β-catenin in the AOM/DSS group compared to that in controls, consistent with activation of the signaling pathway. CU06-1004 treatment significantly decreased β-catenin expression levels (Figure 6C), as well as the expression of *c-Myc*, a key downstream oncogene regulated by β-catenin signaling (Figure 6D). Quantification of β-catenin- and c-Myc-positive cells confirmed a significant reduction in both markers following CU06-1004 administration (Figures 6E,F). These findings suggest that CU06-1004 may contribute to the suppression of tumor growth not only by inhibiting inflammatory responses but also by modulating oncogenic pathways, such as Wnt/β-catenin, in inflammation-induced colon cancer.

## 4 Discussion

**FIGURE 7:**
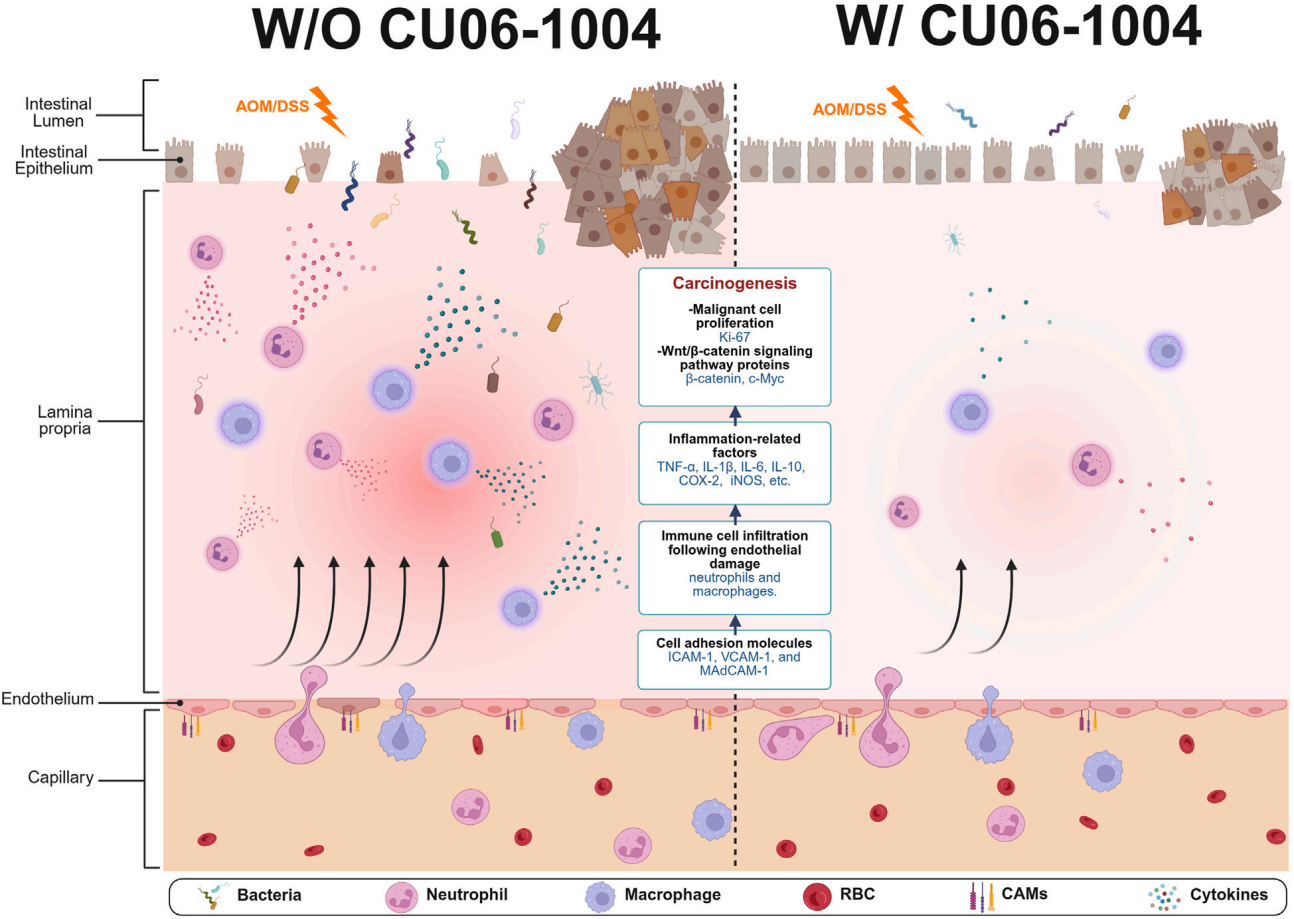
Schematic summary of protective effects of CU06-1004 in chronic colitis and colitis-associated colorectal cancer (CAC) models. CU06-1004 suppresses endothelial adhesion molecules (ICAM-1, VCAM-1, MAdCAM-1) to enhance endothelial barrier function, thereby reducing immune cell infiltration into inflamed colon tissue. This leads to decreased recruitment of neutrophils and macrophages and lowers the production of pro-inflammatory cytokines (TNF-α, IL-6, IL-1β). The reduced inflammatory signaling attenuates oncogenic β-catenin/c-Myc activation, inhibiting tumor cell proliferation and progression. This illustration was created via BioRender.com.

This study provides compelling evidence that CU06-1004, a vascular-protective small molecule, effectively suppresses chronic colonic inflammation and prevents inflammation-driven colorectal tumorigenesis *in vivo*. Using both the DSS-induced chronic colitis model and the AOM/DSS-induced CAC model, we demonstrated that CU06-1004 exerts its biological effects via a multifaceted mechanism by attenuating immune cell infiltration, downregulating pro-inflammatory cytokines, and inhibiting oncogenic β-catenin/c-Myc signaling (Figure 7). These results position CU06-1004 as a promising therapeutic agent capable of targeting both the inflammatory environment and neoplastic transformation in the inflamed colon.

Unlike previous studies that investigated CU06-1004 mainly in the context of acute vascular inflammation (Kim Y. S. et al., 2020; Kim et al., 2023), our findings offer the first evidence of its long-term protective effects in chronic intestinal inflammation and tumorigenesis. To our knowledge, this is the first study to demonstrate that a vascular-stabilizing agent such as CU06-1004 can exert long-term chemopreventive effects in both chronic colitis and CAC models. These findings not only expand the known therapeutic scope of CU06-1004 but also introduce endothelial stabilization as a novel strategy to prevent inflammation-driven colorectal cancer. In the chronic DSS model, CU06-1004 mitigated colitis severity by improving clinical symptoms and maintaining mucosal architecture. Immune cell infiltration contributes to the pathogenesis of CAC by secreting various pro-inflammatory cytokines (Wu et al., 2016). This was mechanistically associated with a significant reduction in the recruitment of neutrophils and macrophages, which play a central role in perpetuating chronic inflammation. Several cytokines produced by immune cells, such as neutrophils and macrophages, are crucial for the immune response in colitis (Vaghari-Tabari et al., 2022). These cytokines mediate an increase in immune cell infiltration, thereby perpetuating a cycle of inflammatory responses and sustaining chronic inflammation (Vaghari-Tabari et al., 2021). Specifically, TNF-α and IL-6, released by inflammatory macrophages and neutrophils, play key roles in the pathogenesis of IBD and CAC (Waldner et al., 2012; Moein et al., 2019). Leukocyte trafficking to inflamed colonic tissue, a hallmark of chronic colitis, is tightly regulated by vascular adhesion molecules. Notably, blockade of these adhesion molecules significantly attenuates colitis severity (Soriano et al., 2000). The suppression of endothelial adhesion molecules, including ICAM-1, VCAM-1, and MAdCAM-1, suggests that CU06-1004 inhibits leukocyte extravasation by reinforcing endothelial junction integrity. CU06-1004 enhances endothelial barrier function via cAMP/Rac1/cortactin-mediated cortical actin ring formation and suppresses NF-κB activation, a key transcription factor regulating adhesion molecule expression (Maharjan et al., 2013; Zhang et al., 2017). Our findings thus extend this mechanism to a chronic inflammatory setting in the gastrointestinal tract.

In the AOM/DSS-induced CAC model, CU06-1004 significantly reduced tumor burden, decreased the incidence of large tumors, and improved histopathological scores. Pro-inflammatory cytokines are key biomarkers of autoimmune inflammation and tissue damage in the colon (Rahat and Shakya, 2016). Inflammatory molecules are involved in the multiple processes associated with colitis-related colon carcinogenesis (Vendramini-Costa and Carvalho, 2012). Some inflammatory molecules directly impact epithelial cells and activate signaling pathways involved in colitis-related colon carcinogenesis (Vaghari-Tabari et al., 2022). In addition, pro-inflammatory cytokines alter the tumor microenvironment, promoting tumor growth in CAC (Jeong et al., 2014). Tumor-associated inflammation, characterized by elevated TNF-α, IL-6, and IL-1β, was effectively suppressed in both colon tissue and systemic circulation. These pro-inflammatory cytokines not only sustain chronic inflammation but also contribute to tumor initiation and progression by activating oncogenic signaling pathways, including NF-κB and STAT3, which in turn stabilize β-catenin signaling (Lee et al., 2009; Luo and Zhang, 2017; Yao et al., 2019). β-catenin, when translocated from the cytoplasm to the nucleus, can function as a transcription factor to activate the expression of its downstream target gene (*c-Myc*) (Vermeulen et al., 2010). β-catenin activation drives the transcription of proliferative and survival genes such as c-Myc and cyclin D1, facilitating the transformation of inflamed epithelium into dysplastic and neoplastic lesions (Bienz and Clevers, 2000; Reya and Clevers, 2005). Our observation that CU06-1004 attenuates β-catenin and c-Myc expression suggests an indirect but functionally significant blockade of this oncogenic axis, likely via upstream modulation of inflammatory cytokine signaling. Importantly, CU06-1004 exerts its anti-tumorigenic effects not by directly targeting epithelial oncogenic drivers, but by stabilizing vascular endothelial function and suppressing pro-inflammatory cytokine production. This upstream targeting disrupts the inflammatory microenvironment that fuels β-catenin-driven tumorigenesis, representing a mechanistically distinct approach from conventional chemopreventive agents.

Notably, CU06-1004 mediates its effects via multiple mechanisms involving vascular protection and modulation of inflammatory signaling, distinguishing it from conventional IBD or CRC therapies. Whereas most chemopreventive strategies in CAC focus on inhibiting epithelial proliferation (Modesto et al., 2022), modulating immune responses (Wen et al., 2024), or reducing oxidative stress (Zhao et al., 2025), CU06-1004 exerts its action by directly stabilizing the vascular endothelium (Kim Y. S. et al., 2020). This vascular-targeted mechanism reinforces endothelial junction integrity, thereby preventing leukocyte extravasation and interrupting the early stages of the inflammatory cascade. Biologics, such as anti-TNF agents (e.g., infliximab, adalimumab) and anti-integrin therapies (e.g., vedolizumab), have shown efficacy but carry risks of immunosuppression and systemic infections (Lautenschlager et al., 2014; Roblin et al., 2020; Barberio et al., 2022). Similarly, JAK inhibitors and IL-23 blockers are emerging strategies, but long-term safety remains a concern (Sandborn et al., 2022). CU06-1004 offers a distinct mechanism by preserving vascular stability and reducing leukocyte infiltration through modulation of the endothelial barrier. Moreover, its ability to suppress tumor-associated inflammatory pathways suggests potential for use not only as a preventative agent in high-risk IBD patients but also as an adjunct to existing anticancer therapies.

While these findings are promising, several limitations should be acknowledged. Although chemically induced disease models in mice are widely accepted, they may not fully replicate the genetic, microbial, and environmental complexity of human IBD-associated colorectal cancer. To address this limitation, future studies will utilize IL-10 knockout mice, which spontaneously develop chronic colitis and more closely mimic the pathophysiological features of human IBD. Further validation is also planned using endothelial cell–specific transgenic models and patient-derived organoid xenografts to enhance the translational relevance of our findings. Complementary to these approaches, detailed cellular-level and spatial molecular analyses may provide deeper insights into the mechanisms by which CU06-1004 remodels the inflammatory tumor microenvironment. Moreover, in this study, CU06-1004 was administered orally at the time of disease induction, demonstrating its preventive effects on inflammation and disease progression. However, clinical interventions typically commence after symptom onset; thus, our early preventive administration model may not fully reflect the clinical scenario. Therefore, future studies should focus on evaluating the therapeutic efficacy of CU06-1004 when administered after established inflammation and tissue damage, that is, following the diagnosis of ulcerative colitis. Such investigations will provide more direct evidence regarding whether CU06-1004 can promote tissue repair through inflammation resolution or delay the progression of colitis-associated colorectal cancer, thereby enhancing its translational potential and informing therapeutic strategies.

In conclusion, this study establishes CU06-1004 as a potent inhibitor of inflammation-driven colorectal tumorigenesis. By reinforcing endothelial barrier function, attenuating immune cell trafficking, suppressing cytokine production, and modulating β-catenin-mediated oncogenic signaling, CU06-1004 interrupts the pathogenic cascade linking chronic inflammation to colorectal cancer. These findings not only extend the therapeutic potential of CU06-1004 but also underscore the importance of vascular modulation as a novel strategy in preventing colitis-associated colorectal cancer.

## 5 Conclusion

CU06-1004 effectively inhibited the progression toward chronic intestinal inflammation and subsequent colitis-associated colorectal cancer by suppressing immune cell infiltration, reducing pro-inflammatory cytokine production, limiting tumor cell proliferation, and downregulating oncogenic proteins such as β-catenin and c-Myc. These findings highlight CU06-1004 as a promising therapeutic candidate for interrupting the inflammatory cascade and preventing inflammation-driven colorectal carcinogenesis.

## Data Availability

The original contributions presented in the study are included in the article/Supplementary Material, further inquiries can be directed to the corresponding author.
