# Supplementary material for: CU06-1004 inhibits the progression of chronic colitis and colitis-associated colorectal cancer by suppressing inflammation
Source: Front Pharmacol. 2025 Oct 22;16:1684870. doi: 10.3389/fphar.2025.1684870 (PMC12586998; doi:10.3389/fphar.2025.1684870)

Supplementary Material

# Supplementary Figures and Tables

## Supplementary Figures


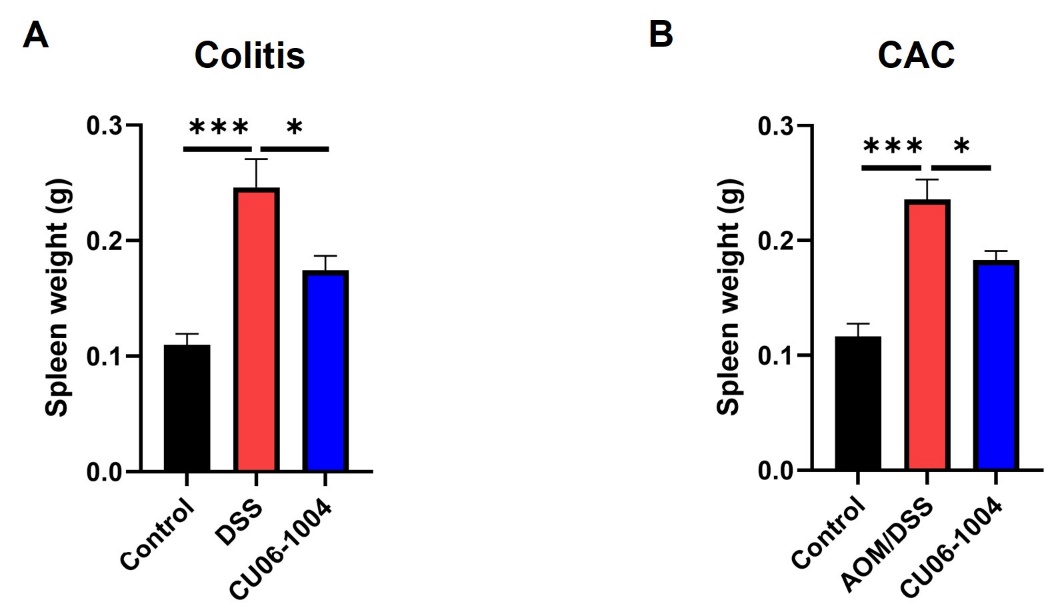


**Supplemental Figure 1. CU06-1004 treatment inhibited spleen enlargement in the mouse model.**

**(A)** Spleen weight was measured when mice with DSS-induced chronic colitis were euthanized. n= 6–8 per group. **(B)** Spleen weight was measured when mice with AOM/DSS-induced colorectal cancer were euthanized. n= 6–7 per group. Data presented as mean ± SEM values; * *P* < 0.05; *** *P* < 0.001.


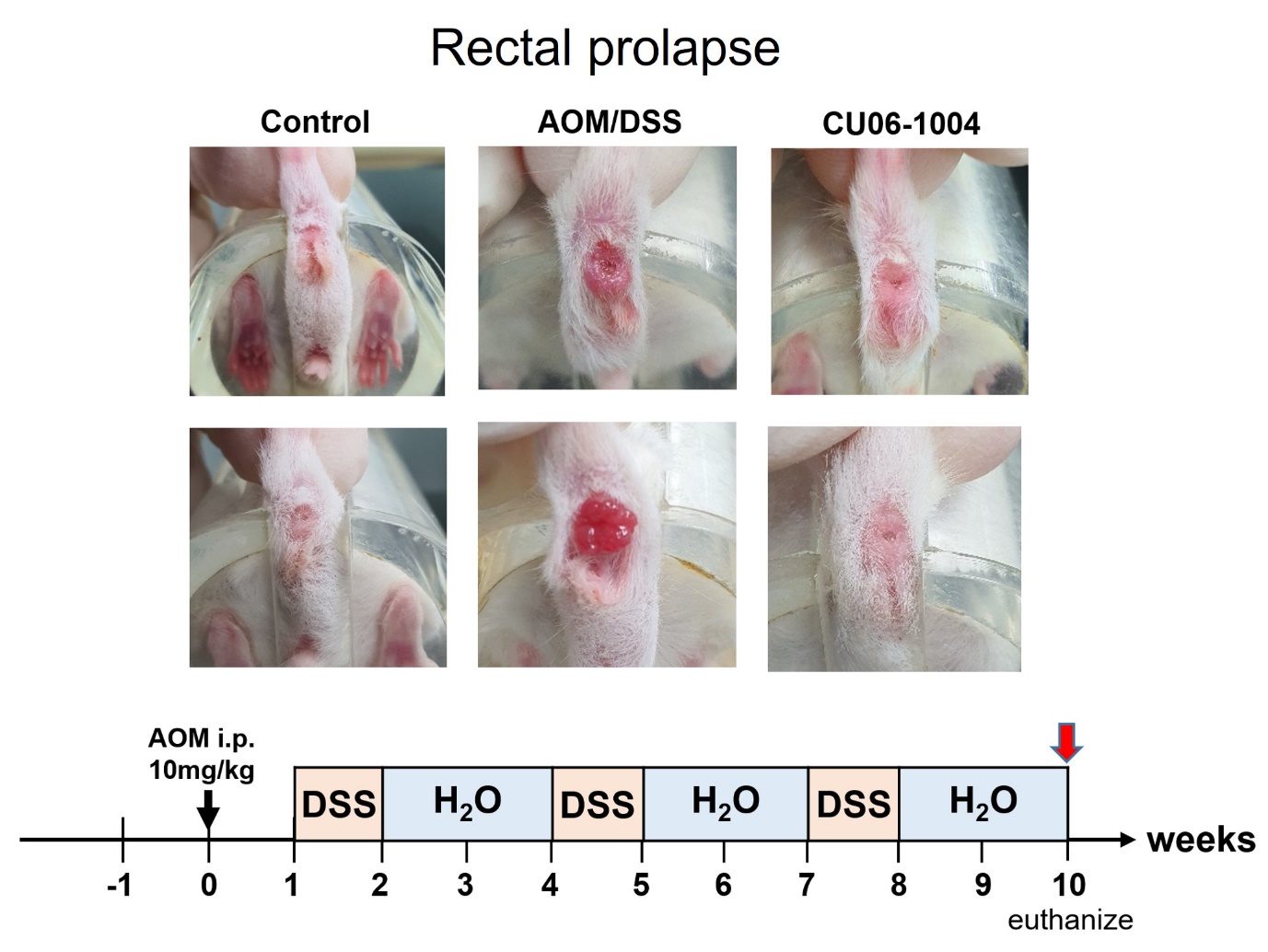


**Supplemental Figure 2. CU06-1004 treatment alleviated rectal prolapse symptoms in the AOM/DSS-induced colorectal cancer mouse model.**

Representative images of rectal prolapse were recorded at the tenth week of the experiment.

## Supplementary Tables

**Supplemental Table 1. Disease activity index (DAI) scores.**


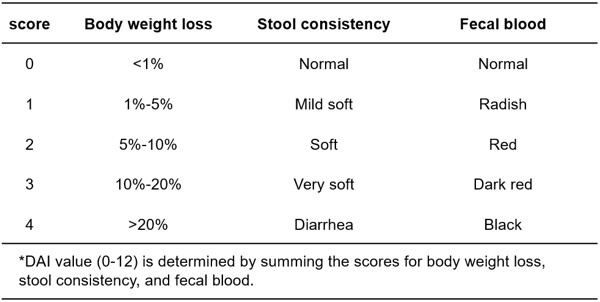


**Supplemental Table 2. Primer sequences for specific genes used in real-time RT-PCR analysis.**


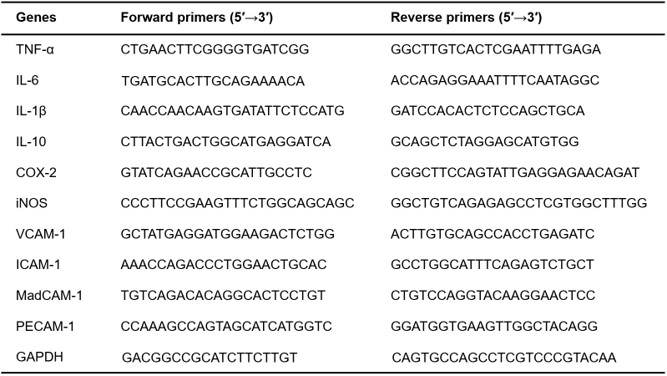

Supplement: Supplementary file 1 [file Supplementaryfile1.docx]
